# Evaluation of the bonding strength of conventional, anatomized, adjustable, and CAD/CAM milled fiberglass posts in weakened endodontically treated roots

**DOI:** 10.1590/0103-644020246103

**Published:** 2024-12-16

**Authors:** Fillipe Mendes Silva, Pedro Henrique Soares Aguiar, Helena Cristina de Assis, Fabiane Carneiro Lopes-Olhê, Jardel Francisco Mazzi-Chaves, Ricardo Gariba Silva, Antonio Miranda Cruz

**Affiliations:** 1 Department of Restorative Dentistry, School of Dentistry of Ribeirão Preto, University of São Paulo (USP), Ribeirão Preto, SP, Brazil.; 2 University of Montes Claros (UNIMONTES), Montes Claros, MG, Brazil.

**Keywords:** root canal filling, fiberglass, adhesion, CAD/CAM

## Abstract

This study evaluated the bond strength (BS) of conventional, anatomized, adjustable, and CAD/CAM fiberglass post systems. Forty maxillary canine roots were weakened, endodontically treated, and divided into four groups (n=10). A slice from each of the three sections (cervical, middle, apical) of the root canal was subjected to the push-out test and the failure pattern was subsequently analyzed. The most apical slice was subjected to analysis of the cementation line thickness by scanning electron microscopy (SEM). Data regarding the BS and cementation line thickness were subjected to the Shapiro-Wilk normality test followed by the variance and Tukey tests. Data failure patterns were expressed as a percentage and submitted to the chi-square test. Conventional fiberglass posts had the lowest BD compared to the other fiberglass posts (p<0.05). All fiberglass posts exhibited a higher percentage of adhesive failures to dentin. The SEM demonstrated a thicker cementation line for the conventional post compared to the other groups (p<0.05), as well as gaps and voids in the cementing material. The anatomized, adjustable, and CAD/CAM milled fiberglass post systems demonstrated a superior BS and adaptation to the root dentin compared to the conventional fiberglass post.

## Resumo

Este estudo avaliou a resistência de união (RU) de sistemas de pinos de fibra de vidro convencionais, anatomizados, ajustáveis ​​e CAD/CAM. Quarenta raízes de caninos superiores foram enfraquecidas, tratadas endodonticamente e divididas em quatro grupos (n=10). Um *slice* de cada terço do canal radicular (cervical, média e apical) foi submetido ao teste de *push-out* e o padrão de falha foi analisado posteriormente. O *slice* mais apical foi submetido à análise da espessura da linha de cimentação por microscopia eletrônica de varredura (MEV). Os dados referentes à RU e espessura da linha de cimentação foram submetidos ao teste de normalidade de Shapiro-Wilk seguido dos testes de variância e Tukey. O padrão de falha foi expresso em porcentagem e submetido ao teste qui-quadrado. O pino convencional apresentou menor RU em comparação aos demais pinos de fibra de vidro (p<0,05). Todos os pinos de fibra de vidro apresentaram maior percentual de falhas adesivas à dentina. A MEV evidenciou linha de cimentação mais espessa para o pino convencional em relação aos demais grupos (p<0,05), além de lacunas e espaços vazios no material restaurador. Os pinos de fibra de vidro anatomizados, ajustáveis e fresados em CAD/CAM demonstraram RU superior e melhor adaptação à dentina radicular em comparação com o pino de fibra de vidro convencional.

## Introduction

Fiberglass posts present favorable aesthetics, no corrosion, and better biomechanical behavior, when compared to metal posts. The physical properties of fiberglass posts, such as the modulus of elasticity, are more similar to those of dentin, which allow a better distribution of forces and reduces the risk of root fracture when compared to other more rigid materials ^1^.

The diameter of the post and thickness of the cement line are important factors to consider, which can affect the bond strength (BS) of fiberglass post cementing systems, since the BS is significantly lower when the resin cement layer is thicker ^2^. A retrospective clinical study reported that posts cemented with thicker material detached more easily than posts with thinner cementing material ^3^.

Teeth with significantly wide canals, or those weakened by endodontic treatment, have led to the introduction of customized or anatomized retainers obtained through a direct technique, which molds the root canal with composite resin associated with a prefabricated fiberglass post ^4^. Customization of the fiberglass post allows for a better adaptation to the prepared space, forming a thin layer of cement, creating favorable conditions for the post retention on the root canal walls, and consequently increasing the BS ^5^. However, studies have demonstrated that customization does not contribute to an improved BS, especially in a short-term analysis, such as in laboratory studies ^6^. One of the disadvantages of manual customization is that the materials used have different biomechanical properties and another interface is added to the cementation line between the prefabricated post and the composite ^7^.

A new intra-radicular retainer with an adjustable fiberglass post system (single adjustable post system - SAP) was developed to achieve a better adaptation of the fiberglass post in root canals. The manufacturer reports that the system functions as a single post that can be adjusted to any situation, such as narrow, medium, or wide canals, without excessive wear on the tooth structure. This post adjusts to the shape of the canal, from apical to cervical, through a conical “sleeve” that surrounds the intra-radicular retainer. The use of SAP resulted in an increase in dentin BS when compared to anatomical posts. The adaptation of the fiberglass post and “sleeve” provides a more homogeneous distribution of the thickness of the luting agent along the entire length of the root ^8^.

Computer-aided design and computer-aided manufacturing (CAD/CAM) has become increasingly popular, proving to be a practical and efficient method for manufacturing prosthesis. This technology is used both in laboratories and dental offices and has been a viable option for composing intra-radicular retainers, especially considering the possibility of milling the post and core ^9^.

Manufacturing customized fiberglass posts with CAD/CAM milling allows the formation of a cement layer with minimal thickness, simplifies the technique, reduces clinical steps, and eliminates the need to adhesively bond the composite resin to build a suitable core for the retention of the restoration, creating a single layer intra-radicular retention system (7, 9).

The CAD/CAM digital post manufacturing workflow provides adaptation parameters within a clinically acceptable range within a shorter time. These posts have demonstrated satisfactory results in terms of fracture resistance. However, specific studies regarding the CAD/CAM digital posts are limited, thus laboratory and clinical studies using this technology are necessary, in which tests evaluating parameters such as clinical longevity and shear strength are conducted ^10^.

The push-out test was employed to assess the BS to root dentin across three different root canal sections (cervical, middle, apical). This method is considered the most suitable for assessing adhesion and accurately representing bonding conditions of the posts in root canals ^11^. Failure pattern was analyzed using a stereomicroscope, and the quality and thickness of the resin cement to the root dentin were analyzed by using photomicrographs obtained through scanning electron microscopy (SEM). Stereomicroscopes are frequently used for this purpose ^12^, while SEM provides high-resolution images at different magnifications, allowing for accurate analysis of cement quality and thickness ^13^.

Given the significant impact of post adaptation and embrittlement on the survival and durability of restorative treatments, and the lack of comprehensive studies on the best approach for rehabilitating weakened teeth, This study aimed to compare the BS of conventional fiberglass posts, anatomized fiberglass posts, adjustable fiberglass posts, and CAD/CAM-milled fiberglass posts to root dentin. Additionally, the study sought to evaluate failure patterns observed after the push-out test, the quality of the adhesive interface formed between root dentin, resin cement, and the fiberglass post, and the thickness of the cementation line. The null hypothesis proposed that all types of fiberglass posts would exhibit similar results regarding bond strength, failure pattern, adhesive interface quality, and cement line thickness.

## Materials and Methods

This study was approved by the Local Research Ethics Committee (CAAE: 66896223.6.0000.5419). The sample size was determined based on a previous study ^13^ and calculated using the ANOVA test in G*Power 3.1.9.7 software (UCLA Advanced Research Computing, Los Angeles, CA, USA). The input parameters were as follows: an effect size of 0.99, a significance level (α) of 0.05, a power (β) of 0.75, and 1 degree of freedom. The calculation indicated that a total sample size of 10 specimens per group would provide an actual power of 0.78.

Maxillary canines recently extracted for therapeutic reasons were collected. The teeth were radiographed to ensure the following inclusion criteria: complete root formation, presence of a single canal, root and canal without curvature, calcifications, resorptions, cracks or previous endodontic treatment. Forty teeth were preselected, rinsed in running water for 24 h, and stored in 0.1% thymol solution at 5°C for up to 30 days.

The root of each specimen was measured using a digital caliper (Digimess, Shiko Precision Gaging Ltd, China) and marked with graphite at a distance of 16 mm from the root tip. Once marked, the teeth were cross-sectioned using a cutting machine (Isomet 1000, Buehler, Lake Forest, IL, USA). The crown portion was discarded, and the root remnant (16 mm) was inserted into an Eppendorf polypropylene tube (Eppendorf Brasil LTDA., São Paulo, SP, Brazil) containing 1 mL of distilled water.

Next, the diameter of the canals was measured in both buccolingual and mesiodistal planes using a digital caliper. Roots with canal diameters larger than the 2-mm posts used in the conventional group (Reforpost n°1, Angelus, Londrina, PR, Brazil) were discarded and replaced with roots meeting the required criterion. Additionally, the amount of dentin around the canal was measured to ensure a minimum 2-mm thickness on the buccal, lingual, mesial, and distal aspects ^14^.

Subsequently, the root canals were irrigated by using a disposable plastic syringe (Ultradent Products, South Jordan, UT, USA) with 2.5 mL of 2.5% sodium hypochlorite (NaOCl) and explored with a K#10 instruments (Dentsply Maillefer, Ballaigues, Switzerland) until the free end of the instrument was visible in the apical foramen. Subsequently, 1.0 mm was subtracted from this measurement to establish the working length (WL), and the preparation was performed using the Logic 2 System files (Easy Produtos Odontológicos, Belo Horizonte, MG, Brazil), driven by an electric motor (VDW Silver, VDW GmbH, Munich, Germany). The root canal was conducted with the initial instrument plus the sequential files up to the surgical diameter corresponding to the last file of the instrumentation, 40.05 (Easy Produtos Odontológicos, Belo Horizonte, MG, Brazil), followed by final irrigation with 2 mL of 17% ethylenediaminetetraacetic acid (EDTA) for 5 min and irrigation with 5 mL of 2.5% NaOCl.

The canals were first dried using Capillary Tips aspiration cannulas and 40.05 absorbent paper cones (Easy Produtos Odontológicos, Belo Horizonte, MG, Brazil). Next, the adaptation of the 40.05 gutta-percha cone (Easy Produtos Odontológicos, Belo Horizonte, MG, Brazil) to the working length (WL) - which corresponds to the last file used in the instrumentation - along with the accessory cones, was verified. This was done by taking digital radiographs in the orthogonal and mesioradial directions to ensure proper obturation using the lateral condensation technique. The AH Plus sealer (Dentsply, Konstanz, Germany) was manipulated according to the manufacturer's instructions and inserted into the root canal with a K-file #40 (Dentsply Sirona Endodontics, Ballaigues, Switzerland) with counterclockwise rotation movements and the cone was greased with sealer and introduced with circular and gradual movement up to the WL. The excess of filling material at the canal entrance was removed with a previously heated Paiva condenser (Golgram, São Caetano do Sul, SP, Brazil). Then, with the gutta-percha still plasticized, cold vertical condensation was performed, with light pressure in apical direction for 5 seconds. The final cleaning of the root canal entrance was performed with a sponge dampened in alcohol, and the absence of voids was checked by means of orto and mesiorradial digital radiographies. The specimens were stored at 37 °C for 72 h for the subsequent preparation of the post space and cementation of the fiberglass post.

First, the specimens were slowly unfilled with a preheated Rhein tip (Golgran, São Caetano do Sul, Brazil) to a depth of 12 mm. They were prepared by the same operator, a specialist in Endodontics, and were then randomly distributed into 4 groups (n=10) according to the restoration protocol: conventional fiberglass post (Reforpost, Angelus, Londrina, Brazil); anatomized fiberglass post with composite resin (Reforpost, Angelus, Londrina, Brazil; Filtek Z350 XT, 3M ESPE, St. Paul, MN, USA); adjustable fiberglass post (Splendor SAP, Angelus, Londrina, Brazil); and fiberglass post milled in CAD/CAM system (Fiber Cad Post and Core, Angelus, Londrina, Brazil).

The roots were inserted into aluminum molds (16 x 16 x 32 mm) and embedded in acrylic resin (Jet, Clássico, São Paulo, Brazil), leaving 2 mm of the cervical length of the roots exposed above the upper limit in the acrylic resin block, thus obtaining the specimens.

The embrittlement was prepared by following the drill sequence: 718PM, 730PM, and 720PM (KG-Sorensen, São Paulo, SP, Brazil), in extensions of 8, 6 and 8 mm, respectively. The preparations were performed by using a low-speed straight piece (Kavo Kerr, São Paulo, SP, Brazil).

In both the conventional and anatomized groups, the root canal walls were prepared to a length of 12 mm using Largo reamer drills numbered 1, 2 and 3 (Dentsply Sirona, São Paulo, SP, Brazil), with the n°3 drill being compatible with the diameter of the fiberglass post Reforpost n°1 ^15^. In the CAD/CAM group, the root canals walls were also prepared to a length of 12 mm using Largo reamer drills numbered 1, 2 and 3, as this system does not require a specific drill ^16^. For the adjustable post group, a universal drill from the Splendor Kit recommended by the manufacturer was used, which was coupled to a micromotor (Dabi Atlante, Ribeirão Preto, Brazil) ^12^. The removal of gutta-percha from the root canal walls was examined using a dental operating microscope (DF Vasconcelos, São Paulo, SP, Brazil) at 6.0x magnification. The presence of the remaining 4 mm of filling material was then confirmed through digital radiography. The canals were irrigated with distilled water, aspirated with a suction cannula, and dried with 40.05 absorbent paper tips.

Prior to cementation, the conventional posts were cleaned with 70% alcohol for 10 min, dried with air jets, coated with a silane (Angelus Ind Prod Odontológicos S/A, Londrina, PR, Brazil) layer, and left to dry for 60 s ^13^. Resin cement RelyX U200 Automix (3M ESPE, St. Paul, MN, USA) was inserted into the root canal with applicator tips, and then onto the surface of the fiberglass posts, which were cemented into the root canals by digital pressure. While maintaining the post in position, the excess material was removed. Photoactivation was performed for 60 s with a light-curing device (Elipar**,** 3M ESPE, St. Paul, MN, USA) (1470 mW/cm², wavelength 430-480 nm, continuous mode) ^17^. The light-curing tip was placed in direct contact with the coronal end of the fiberglass post, allowing the light to be transmitted into the root canal through the post itself. Prior to use, irradiance was measured with a radiometer (L.E.D. Radiometer by Demetron, Kerr Corporation, Middleton,WI, USA).

For the confection of anatomized posts, the root canal was first isolated using a water-soluble gel (Johnson and Johnson, New Brunswick, New Jersey, USA), which was applied with a brush (Faber Castell, São Carlos, SP, Brazil). The posts were cleaned with 37% phosphoric acid (Ultra Etch, Ultradent, Salt Lake City, Utah, USA) for 1 min, then washed with water, dried with air jets, and coated with a silane layer. The posts were left to dry for 60 s. A layer of light-curing adhesive (Single Bond, 3M ESPE, St. Paul, MN, USA) was then applied to the post and photoactivated for 20 s using a light-curing device (Elipar, 3M ESPE, St. Paul, MN, USA). A portion of composite resin (Filtek Z350 XT, 3M ESPE, St. Paul, MN, USA) was placed over the post. This setup, without light curing, was inserted into the root canal, and photoactivation was performed on one side for 5 s. This setup was then removed from the canal and photoactivated for 60 s. Before cementation, the post was cleaned with 70% alcohol for 10 min, dried with air jets, and coated with a silane layer. It was left to fry for 60 s. RelyX U200 Automix resin cement (3M ESPE, St. Paul, MN, USA) was inserted directly into the root canal using the special self-mixing tip and photoactivated for 60 s using a light-curing device ^14^.

The adjustable posts were also cleaned with 70% alcohol for 10 min, dried with air jets, coated with silane on the cylindrical universal post and conical sleeve, and left to dry for 60 s. Relyx U200 Automix resin cement (3M ESPE) was inserted into the root canal and photoactivation was performed similar to the previous group ^12^ **.**

To manufacture the fiberglass post using the CAD/CAM system, the root canal was molded using the direct technique by relining with acrylic resin and Pinjet (Angelus, Londrina, Brazil). The Pinjet post was then scanned using an inEos X5 scanner (Dentsply Sirona, São Paulo, SP, Brazil). The fiberglass post was milled using a Ceramill Motion 2 milling machine (Amann Girrbach Brasil LTDA, Curitiba, PR, Brazil) in a fiberglass block (Fiber Cad Post and Core, Angelus, Londrina, Brazil), with successive changes of Roto t1, t2, and t3 drills (Amann Girrbach Brasil LTDA**,** Curitiba, PR, Brazil) ^17^. Cleaning, disinfection, and cementation of the fiberglass posts followed the same protocol as in the previous groups.

The slices for the push-out test and SEM analysis were obtained by placing the roots on acrylic resin plates with the long axis parallel to the surface of the plate and fixed with hot glue. The roots were sectioned perpendicular to their long axis in the mesial-distal direction by using a 0.3 mm-thick diamond blade (South Bay Technology, San Clement, CA, USA) at 350 rpm and weighing 75 g under constant refrigeration in a precision cutting machine (Isomet 1000, Buehler, Lake Forest, IL, USA). The first slice from each third of the root canal was used for the push-out test, and the second, more apical, was used for the SEM analysis.

The slices were positioned on stainless steel metal bases attached to the bottom of the universal testing machine (model 2519-106; Instron, Canton, MA, USA). Following the diameters of the restorative material in the three sections (cervical, middle, and apical) of the roots, we used metal bases with holes measuring 1.2, 1.5, and 2.5 mm in diameter in their central portion, and metal rods with active tips measuring 0.3, 0.4, 0.5, 0.6, 0.7, 0.8, 0.9, 1, 1.1, 1.2, 1.5, 2, and 2.5 mm in diameter, respectively. The specimens were positioned in the same direction as the hole in the metal base, with the cervical side facing downwards. The rods were attached to the top of the testing machine and positioned over the material. Subsequently, the testing machine was operated at a constant speed of 0.5 mm/min until reaching the maximum tension required to move the material. This methodology ensured a precise and reproducible alignment, and the rods used were aligned in the material and did not contact the dentin walls.

The force required for displacement was measured in Newtons (N). To calculate the BS, the resulting force was converted into Megapascals (MPa) and divided by the lateral area of the intracanal material. For the exact calculation of the lateral adhered area (SL), the geometric aspect of the intracanal material (resin cement + fiberglass post) was considered according to the level of the cut to obtain the slices. Prior to the push-out test, the thickness (h) of each slice was measured with an electronic caliper. The smaller radius (apical side) and the larger radius (cervical side) were assessed using Leica M165C stereomicroscope (Leica Microsystems, Mannheim, Germany) and the LAS v4.4 software (Leica Microsystems). The radius of the restorative material was determined with the Circle Tool (Three Point Type) under the stereomicroscope. This tool enabled accurate measurement of the radius by marking three points on the adhesive interface.

Thus, the cement adhesion area (in mm²) was calculated using the formula of the lateral surface area (LSA) as follows: π (R+r) √(h^2+(R-r)²). Here, R is the radius of the fiberglass post and resin cement in its coronal portion, r is the radius of the fiberglass post and resin cement in its apical portion, and h is the height/thickness of the cut. Based on this data, the BS was calculated in MPa by dividing the force required to detach the fiberglass post by its lateral area (BS=F/LSA).

To analyze the type of failure, the sections were assessed using a Leica M165C stereomicroscope (Leica Microsystems) with a 25x magnification and LAS v4.4 software (Leica Microsystems). The assessment was conducted by an experienced examiner. The failures observed were determined in percentages and classified as follows: a) Adhesive to dentin: absence of the resin cement on the dentin walls. b) Adhesive to post: presence of the resin cement on the dentin walls. c) Adhesive to resin: post detached from the resin. d) Mixed: presence of the resin cement on the dentin walls and around the fiberglass post. e) Cohesive to dentin: fracture in dentin. f) Cohesive to the fiberglass post: fracture in fiberglass post.

SEM analyses were performed to check for possible flaws in the restorative material at each of the three sections (cervical, middle, and apical) of the root. One slice from each third was designated for the SEM analyses, resulting in 15 slices (3 slices x 5 teeth) for each of the 4 tested groups, totaling 60 slices (15 slices x 4 groups). The slices were sanded, dehydrated, and coated with gold-palladium alloy (30 nm-thick) using a Desk II Denton Vacuum metallizer (Moorestown, NJ, USA) under a vacuum. The analysis was performed using a scanning electron microscope (JSM6610LV; JEOL, Tokyo, Japan) operating at 20 kV with the aid of an SEM Control User Interface v.3.06 software. Images of each of the three sections of the root for each group were obtained at a magnification of 40x.

**Figure 1:**
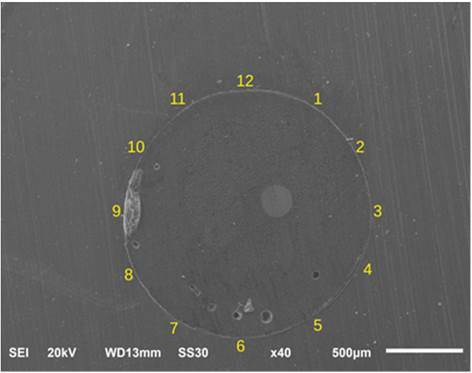
Illustration of the methodology for measuring the thickness of the cementation line at 12 points at the interface between root dentin and glass fiber post.

The ImageJ software (National Institutes of Health, Bethesda, Maryland, USA) was used to analyze the thickness of the cementation line in um. A total of 12 thickness measurements were obtained at points similar to the numbers on a clock. Finally, the mean values for each of the three sections of the root were measured and statistically analyzed (Figure 1).

A statistical analysis was performed using the Statistical Package for the Social Sciences (SPSS) software (IBM Corp, Armonk, NY, USA), version 18.0 for Windows with a probability level fixed at 95% for all tests. The bond strength and resin cement line thickness data were initially subjected to the normality (Shapiro-Wilk, p>0.05) and homogeneity of variance (Levene, p>0.05) tests. Once a normal distribution was confirmed, the two-way ANOVA test was used, followed by Tukey’s complementary test. Data regarding the failure pattern was expressed as a percentage and associated with the post factors (conventional, anatomized, adjustable, and CAD/CAM milled) and three sections of the root canal (cervical, middle, and apical) using the chi-squared test. The power test was conducted using G*Power 3.1.9.7 (UCLA Advanced Research Computing, Los Angeles, CA, USA), considering a significance level (α) of 0.05 and a power (β) of 0.9.

## Results

### Bond strength analysis

The two-way ANOVA test revealed a significant interaction between the type of fiberglass posts and the root thirds (p<0.05). Conventional and CAD/CAM posts did not show significant differences across the root thirds (p>0.05). However, for Anatomized and Adjustable SAP posts, the highest bond strength values were observed in the apical third (p<0.05).

No significant differences were found between the fiberglass posts in the cervical third (p>0.05). In contrast, for the middle and apical thirds, Anatomized, Adjustable SAP, and CAD/CAM posts exhibited the highest bond strength values (p<0.05), with no significant differences among them (p>0.05). The Conventional post showed the lowest bond strength values compared to the other posts (p<0.05) (Table 1).

**Table 1:** Bond strength means and standard deviation, in megapascals (MPa), of the restorative material to dentin in the cervical, middle and apical thirds for the different types of fiberglass posts (n=10).

Different types of fiberglass post				
Root third	Conventional	Anatomized	Adjustable SAP	CAD/CAM
Cervical	1,07 ± 0,71 Aa	2,92 ± 1,53 Ba	2,03 ± 0,86 Ba	2,15 ± 1,36 Aa
Middle	1,01 ± 0,46 Ab	3,81 ± 1,92 Ba	2,59 ± 0,99 Ba	2,79 ± 1,84 Aa
Apical	1,37 ± 0,69 Ab	4,82 ± 2,18 Aa	3,82 ± 2,11 Aa	3,43 ± 2,58 Aa
Mean	1,15 ± 0,6 b	3,85 ± 1,99 a	2,81 ± 1,58 a	2,79 ± 1,99 a

Different uppercase letters indicate a significant difference between rows and different lowercase letters indicate a significant difference between columns (Tukey’ test, p<0.05).

The power of the test exhibited a value of 0.966.

### Failure pattern analysis

The chi-squared test did not reveal a significant association between failure mode type and root thirds (p>0.05) (Table 2). However, a significant difference was observed between failure mode type and the type of fiberglass post (p<0.05).

**Table 2:** Failure mode type after push-out test for the different types of fiberglass posts (percentage values) (n=10).

Failure type					
Root third	Adhesive in dentin	Adhesive in post	Adhesive in resin	Mixed adhesive	Cohesive in dentin
Cervical	31 (77.5)	5 (12.5)	3 (7.5)	0 (0.0)	1 (2.5)
Middle	37 (92.5)	1 (2.5)	1 (2.5)	0 (0.0)	1 (2.5)
Apical	33 (82.5)	4 (10.0)	1 (2.5)	1 (2.5)	1 (2.5)

The power of the test exhibited a value of 0.926.

Overall, all fiberglass posts exhibited a higher percentage of adhesive failures in the dentin type (p<0.05). The Anatomized post showed a higher percentage of adhesive failures in the resin type (p<0.05), while CAD/CAM posts exhibited a higher percentage of adhesive failures in the post type (p<0.05) (Table 3).

**Table 3:** Failure mode type after push-out test for the different types of fiberglass posts (percentage values) (n=10) (p<0.05).

Types of fiberglass post				
Failure type	Conventional	Anatomized	Adjustable SAP	CAD/CAM
Adhesive in dentin	28 (93.3)	23 (76.7)	29 (96.7)	21 (70.0)
Adhesive in post	2 (6.7)	1 (3.3)	1 (3.3)	6 (20.0)
Adhesive in resin	0 (0.0)	5 (16.7)	0 (0.0)	0 (0.0)
Mixed adhesive	0 (0.0)	0 (0.0)	0 (0.0)	1 (3.3)
Cohesive in dentin	0 (0.0)	1 (3.3)	0 (0.0)	2 (6.7)

The power of the test exhibited a value of 0.950.

### Adhesive interface thickness analysis

The two-way ANOVA test demonstrated a significant interaction between the type of fiberglass post and root thirds (p<0.05). Conventional posts exhibited a thicker cementation line in the cervical third compared to the middle third (p<0.05), and the middle third had a thicker cementation line than the apical third (p<0.05). Anatomized and CAD/CAM posts did not show significant differences in thickness between the root thirds (p>0.05). The Adjustable SAP post had a thicker cementation line in the cervical third compared to the middle and apical thirds (p<0.05), although there were no significant differences between the middle and apical thirds (p>0.05).

For the cervical and middle root thirds, the Conventional post had the thickest cementation line (p<0.05). In the apical root third, Conventional, Adjustable SAP, and CAD/CAM posts all exhibited the thickest cementation lines (p<0.05), with no significant differences among them (p>0.05).

Overall, the Conventional post had the thickest cementation line compared to the Adjustable SAP and CAD/CAM posts (p<0.05), which were similar to each other (p>0.05). The Adjustable SAP and CAD/CAM posts also had thicker cementation lines compared to the Anatomized post (p<0.05) (Table 4).

**Table 4:** Thickness means and standard deviation, in micrometers (μm), of resin cement according to the root thirds (cervical, middle and apical) of the fiberglass post and total mean.

Different types of post				
Root third	Conventional	Anatomized	Adjustable SAP	CAD/CAM
Cervical	580,0 ± 162,0 Aa	73,7 ± 41,9 Ab	219,0 ± 79,1 Ab	138,0 ± 57,9 Ab
Middle	295,0 ± 81,9 Ba	32,1 ± 10,8 Ab	44,9 ± 38,2 Bb	103,0 ± 48,2 Ab
Apical	190,0 ± 113,0 Ca	46,9 ± 19,2 Ab	113,0 ± 54,9 Ba	154,0 ± 76,1 Aa
Mean	355,0 ± 205,0 a	50,9 ± 31,9 c	125,0 ± 93,0 b	132,0 ± 64,3 b

Different uppercase letters indicate a significant difference between rows and different lowercase letters indicate a significant difference between columns (Tukey’ test, p<0.05).

The power of the test exhibited a value of 0.993.

**Figure 2:**
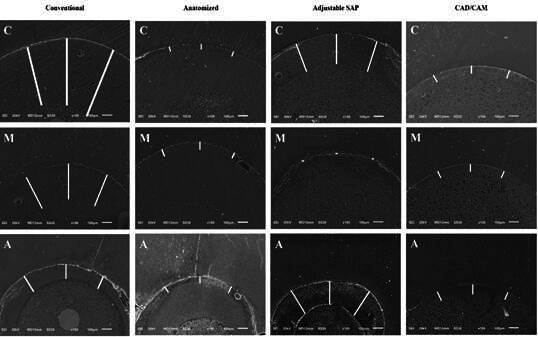
Photomicrographs of the qualitative assessment of resin cement thickness at the root dentin/resin cement/fiberglass post interface of the cervical (C), middle (M) and apical (A) thirds (x100) in all fiberglass posts. The white line indicates the resin cement thickness.

The statistical differences observed in the evaluation of the fiberglass post are corroborated by the qualitative analysis of the SEM images (Figure 2). The photomicrographs reveal a larger cement line in the Conventional post, particularly in the cervical (C) and middle (M) thirds, while the other groups displayed a thinner resin cement line.

**Figure 3:**
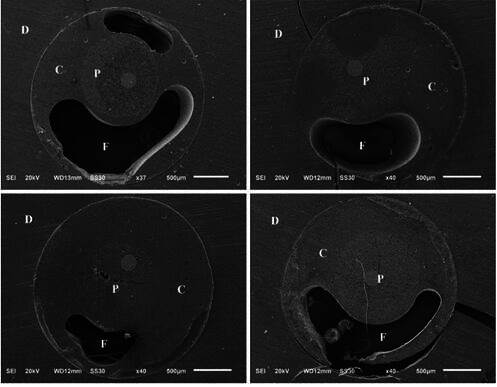
Photomicrograph showing flaws (F) at the resin cement (C), glass fiber post (P) and root dentin (D) interface, in the conventional glass fiber post group.

A higher percentage of gaps was observed in the adhesive interface between the fiberglass post, resin cement, and root dentin for the Conventional post, indicating a lack of cementing material (Figure 3). This group exhibited significant maladjustment, with noticeable gaps and voids

**Figure 4:**
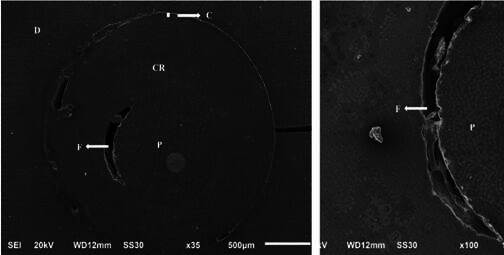
Photomicrograph demonstrating a small band of cement (C), in addition to a flaw (F) at the interface between composite resin (CR) and anatomized fiberglass post (P).

Gaps at the fiberglass post/composite resin interface were also observed for the Anatomized post in the SEM images (Figure 4). These failures are attributed to operational errors during the creation and anatomization of the pin, highlighting the delicate nature of this technique, which requires precise training for successful execution.

**Figure 5:**
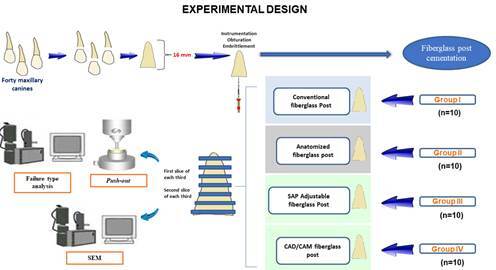
Flowchart illustrating the distribution of groups and the subsequent analyses. A total of 40 teeth were divided into four groups: conventional, anatomized, adjustable, and CAD/CAM fiberglass posts. For each group, the first slice of each third was used for the push-out test and failure pattern analysis (n=10), while the second slice of each third was utilized for SEM analysis (n=5).

## Discussion

The integrity of the adhesive interface between the resin cement and dentin wall depends on factors such as the interaction between the adhesive system and resin cement, shape, and composition of the fiberglass post, as well as the shape and diameter of the three root canal sections (cervical, middle, and apical) ^18^. Therefore, the proper selection of intra-radicular posts is irrelevant for the adhesion process, as the post must have good adaptation to the root canal, forming a thin cementation line ^5^ ^,^ ^19^.

This study evaluated the bond strength and thickness of the resin cement at the post interface in the cervical, middle, and apical sections of conventional, anatomized, adjustable, and CAD/CAM milled fiberglass posts. The results indicated that anatomized, adjustable, and CAD/CAM posts exhibited higher bond strength compared to conventional posts. However, no significant difference was found among these three types of posts. This finding is consistent with a recent study that assessed conventional, anatomized, adjustable, and CAD/CAM milled fiberglass posts cemented to flared root canal dentin with RelyX U200, the same resin cement used in this in vitro study ^20^. Additionally, the posts evaluated in this study exhibited thinner cementation lines compared to conventional posts, leading to the rejection of the null hypothesis.

Among the posts evaluated, anatomized posts exhibited the highest bond strength values compared to conventional fiberglass posts. This result is consistent with a previous study that found anatomized posts had greater bond strength when cemented with RelyX ARC and RelyX Unicem, which are conventional and self-adhesive resin cements, respectively ^21^. The enhanced performance of anatomized posts is attributed to their customization with composite resin, which improves adaptation to the root canal and results in a thin cementation line (50.9 µm) ^5^ ^,^ ^19^. SEM images confirmed that anatomized posts had the thinnest cementation line, while conventional posts had the thickest (Figure 2).

Adjustable posts also demonstrated higher bond strength values to conventional fiberglass posts, which can be explained by their structural composition ^22^. The SAP adjustable post, which features a conical fiberglass “sleeve” with a slit along its long axis, adapts to the space between the post and the root canal. This design facilitates micromechanical adhesion and mechanical retention ^23^. The adaptability of the adjustable post creates a more homogeneous interface between the post, “sleeve”, and cement ^23^. Previous studies have also highlighted the advantages of adjustable posts over conventional fiberglass posts ^12^ ^,^ ^22^ ^,^ ^23^. For instance, Alves dos Santos et al. (2023) reported that adjustable posts had higher bond strength values compared to conventional fiberglass posts when both were cemented with RelyX U200, the same resin cement used in this study.

CAD/CAM-milled fiberglass posts also showed high bond strength values, as supported by previous research ^24^ ^,^ ^25^. These posts adapt well even in wide canals, reducing the thickness of the cement layer and minimizing gaps ^26^ ^,^ ^27^. In this study, the cement thickness was approximately 132 µm, similar to previous reports ^27^. Previous studies have shown satisfactory results for both adjustable and CAD/CAM-milled posts under tensile strength testing, indicating similar performance ^22^. The CAD/CAM system’s advantage lies in creating a post and core as a single piece, eliminating interfaces between the fiber post and composite resin, leading to stronger resin cement bonds ^22^.

The conventional fiberglass post had the lowest bond strength values. Photomicrographs revealed an irregular and thick resin cement layer with numerous adhesive failures and gaps (Figure 3), which were not observed in the other groups*.* The low bond strength values and failures are likely due to polymerization contraction caused by the broad resin cement layer, which created structural discontinuities at the dentin/cement and cement/post interfaces, leading to bubbles and gaps that reduced post retention in the root canal ^28^. Previous research also suggests that the geometric configuration of the root canal can increase the C factor, which refers to polymerization contraction stress within the root canal ^29^.

Adhesive failures in dentin were the most prevalent across all posts following the push-out test. Images from the compression tests indicated that most specimens lacked resin cement on the walls, which is consistent with data from previous studies ^22^. This observation suggests that the bond between the post and resin cement is stronger than that between the cement and dentin wall. This finding is crucial for the effective adhesive cementation of fiberglass posts ^27^.

Anatomized, adjustable, and CAD/CAM-milled fiber posts were compared due to their similar properties and the limited number of relative studies available in the literature. All three types of posts are suitable for use in wide canals with extensive coronal destruction. However, from a clinical perspective, anatomized and adjustable posts can be placed in a single session, whereas CAD/CAM fiber posts require two steps, one of which is performed in the laboratory. Consequently, a limitation of this in vitro study is the method used for manufacturing CAD/CAM-milled fiberglass posts. Specifically, posts made from acrylic resin were scanned to create the final post and core. While this method is viable, it demands additional time and expertise. A more efficient approach would involve direct intracanal scanning to produce a digital post model for CAD/CAM milling.

Another limitation of the study is the lack of bond strength values after artificial aging. It is crucial to assess whether storage time or exposure to thermal stress affects the bond strength of the adhesive interface to root dentin. Literature suggests that degradation of adhesive components may decrease bond strength ^13^ ^,^ ^30^.

Based on the results of this in vitro study and the employed methodology, anatomized, adjustable, and CAD/CAM-milled fiberglass posts appear to be promising alternatives for the rehabilitation of weakened teeth. These posts demonstrated higher bond strength values, a thinner cementation line, and fewer gaps compared to conventional fiberglass posts. While the study indicates the superiority of these posts, further research is necessary. Dentistry involves diverse clinical situations, each requiring different methods. Variations in root canal morphology, remaining tooth tissue, and occlusal forces could all influence post selection. These variables should be explored in future studies.
